# Exploration of modern contraceptive methods using patterns among later reproductive-aged women in Bangladesh

**DOI:** 10.1371/journal.pone.0291100

**Published:** 2024-04-01

**Authors:** Md. Shohel Rana, Shimlin Jahan Khanam, Md. Badsha Alam, Md. Tahir Hassen, Md. Iqbal Kabir, Md. Nuruzzaman Khan

**Affiliations:** 1 Department of Population Science, Jatiya Kabi Kazi Nazrul Islam University, Trishal, Mymensingh, Bangladesh; 2 Centre for Women’s Health Research, Faculty of Health and Medicine, The University of Newcastle, New South Wales, Australia; 3 Climate Change and Health Promotion Unit (CCHPU), Health Services Division, Ministry of Health and Family Welfare, Dhaka, Bangladesh; 4 Department of Disaster Science and Climate Resilience, University of Dhaka, Dhaka, Bangladesh; Health Sciences, Arnavutkoy State Hospital, TURKEY

## Abstract

**Background:**

With the rapid increase in the number of women in their later reproductive years (aged 35 and above) in the present decade, the concern surrounding their contraceptive considerations has reached a critical point of importance. This study aims to examine the trends and determinants of modern contraceptive uptake among later reproductive-aged women in Bangladesh.

**Methods:**

A total of 17,736 women aged 35 and above were included in the analysis, utilizing data from three consecutives Bangladesh Demographic and Health Surveys conducted in 2011, 2014, and 2017–18. The outcome variable was the uptake of modern contraceptive methods (yes or no). The explanatory variables encompassed survey years, individual characteristics of the women, as well as characteristics of their partners and the community. Multilevel logistic regression model was used to explore the association of the outcome variable with explanatory variables.

**Results:**

We found that approximately 54% of women aged 35 and more do not use modern contraceptive methods, and there have been no significant shifts in their usage observed over the survey years. Compared to women aged 35–39, women aged 40–45 (aOR = 0.53, 95% CI: 0.49–0.57) and 45–49 (aOR = 0.24, 0.22–0.26) reported lower likelihoods of modern contraceptive method uptake. Higher education correlated with increased uptake of modern contraceptive methods (112%-142%), while partner’s education showed a negative association. Later reproductive-aged women in richer (aOR = 0.83, 95% CI: 0.74–0.94) and richest (aOR = 0.76, 95% CI: 0.66–0.88) quintiles reported lower uptake of modern contraceptive methods compared to their counterparts in the poorest quintile. Later reproductive-aged women in Dhaka (aOR = 1.22, 95% CI: 1.07–1.38) and Rajshahi (aOR = 1.37, 95% CI: 1.19–1.59) regions had higher uptake of modern contraception than those residing in the Barishal division. Modern contraceptive methods uptake was 1.22 times higher among women who reported exposure to mass media and 1.19 times higher among women who reported engagement in paid work compared to among women who reported no exposure to mass media and participation in no formal work, respectively. Modern contraceptive methods uptake was 43% higher (aOR = 1.43, 95% CI: 1.32–1.55) in women with more than 2 children compared to those with ≤2 children.

**Conclusion:**

The study highlights no significant change in modern contraception uptake among later reproductive-aged women in Bangladesh. This raises concerns about the elevated risk of unintended pregnancies and shorter birth intervals, emphasizing the need for targeted interventions to address the specific needs and preferences of this demographic.

## Introduction

The ongoing public health challenges in low- and middle-income countries (LMICs) are major contributor to the higher occurrence of unintended pregnancy, short inter-pregnancy intervals, and a higher rate of pregnancy terminations [1–3]. Access to modern contraceptive methods (e.g., pills, intra-uterine devices (IUDs), and implants) than non-use of contraceptive methods or use of traditional contraceptive methods (e.g., day counting, withdrawal) can effectively address these issues by empowering women to exercise control over their reproductive choices, enabling them to space pregnancies and plan the timing of childbirth [4, 5]. This, in turn, contributes to healthier birth outcomes and reduces the risk of complications associated with closely-spaced pregnancies [6, 7]. Moreover, by addressing them, modern contraceptive methods work as key drivers to reduce maternal mortality [8]. Additionally, the utilization of modern contraceptive methods is linked to a decrease in high-risk pregnancies, such as those among adolescents and older women, resulting in lower rates of preterm births and low birth weights [4]. The use of modern contraceptives methods also facilitates family planning, enabling couples to make informed decisions about the number of children they wish to have, thus aiding in the optimization of maternal and child health resources [9]. These interventions collectively contribute to achieving United Nations Sustainable Development Goals (SDG) 3, which aims to ensure healthy lives and promote well-being for all, and SDG 5, which targets gender equality and women’s empowerment [10, 11]. The contributions of modern contraceptive methods to reducing maternal mortality (70 per 100,000 live births in LMICs) and child mortality (12 and 25 per 1,000 live births for neonatal and under-five mortality in LMICs, respectively), which Bangladesh and the global community are currently trying to achieve as part of the SDGs, are also recognized worldwide [12, 13]. This is even more significant in Bangladesh, with a stagnant rate of maternal mortality (153 per 100,000 live births) and child mortality (16 and 31 per 1,000 live births for neonatal and under-five mortality, respectively) over the years [14].

The focus on contraceptive practices has traditionally centered on women in their prime reproductive years, i.e women aged <35 years. However, as demographics shift and societal dynamics evolve, an emerging demographic subset is garnering increased attention worldwide–later reproductive-aged women (aged 35 years or more) [15, 16]. In LMICs, including Bangladesh, this age-specific phenomenon has even more compelling dimension due to demographic transitions, resulting in a larger number of female population within this age range [17, 18]. For instance, according to the 2022 census in Bangladesh, approximately 18.70% of women aged 35–49 years make up the total women population, and this percentage is projected to increase to 40% by 2030 [19]. Furthermore, considering the rising trend of delayed childbearing and changing family dynamics in LMICs, the implications of contraceptive utilization among later reproductive-aged women become increasingly pertinent [9, 20]. Women in this age group might be at an increased risk of medical comorbidities and complications during pregnancy, making the adoption of effective contraception a pivotal consideration for their well-being. However, later reproductive-aged women in LMICs may encounter distinct challenges when seeking contraception [21]. Cultural expectations surrounding fertility and family structure might influence their decisions, potentially leading to overlooked reproductive health concerns [1, 13]. Additionally, limited healthcare infrastructure and resources in LMICs, along with their focus to include younger women rather than later reproductive-aged women, can hinder access to appropriate contraceptive methods and comprehensive reproductive healthcare services for this demographic [3, 8]. Therefore, gaining a comprehensive understanding of the factors influencing contraceptive practices among later reproductive-aged women and how these practices have changed over the years is of paramount importance [22].

Despite its critical importance, this issue remains largely unexplored in LMICs, including Bangladesh. Existing studies have primarily focused on either young women or the entire reproductive-aged female population and examine socio-demographic factors associated with contraception use [4, 5, 23–25]. As a result, the dynamics of contraceptive methods uptake among later reproductive-aged women remains unknown. This is a significant concern, particularly in Bangladesh, where population momentum results in a larger number of women entering the 35 and more age group each year, surpassing the number of women entering reproductive age [26, 27]. This insufficient attention might be linked with the stagnation of contraception use rate that Bangladesh has witnessed over the years [9, 28]. To address this gap, the current study aims to explore the contraceptive dynamics among later reproductive-aged women in Bangladesh and assess how these dynamics have evolved across different survey periods. Furthermore, the study seeks to identify factors associated with modern contraceptive methods use among these later reproductive-aged women.

## Methods

### Data source and sampling

The data were derived from three consecutives Bangladesh Demographic and Health Surveys (BDHS) conducted in 2011, 2014, and 2017–18. Briefly, these surveys were conducted as part of the Demographic and Health Survey Program of the USA, similar to surveys conducted in other 89 LMICs. The financial support for these surveys was provided by the USAID with the aim of offering up-to-date information on maternal and child health indicators in LMICs. Detailed information about the methodologies employed in the BDHS can be located in the corresponding survey reports [29–31]. In summary, these surveys encompassed nationally representative samples of women within the reproductive age bracket (15–49 years), selected through a two-stage stratified random sampling process. In the initial stage, 600 enumeration areas (clusters) for the 2011 and 2014 surveys, and 672 enumeration areas for the 2017–18 survey, were randomly chosen as primary sampling units, based on the National Population and Housing Census conducted in 2011 by the Bangladesh Bureau of Statistics. During the subsequent stage, an average of 30 households per enumeration area were selected via systematic random sampling, culminating in a total of 17,964 households in 2011, 17,989 households in 2014, and 20,160 households in 2017–18. The interviews were carried out in 17,141 households (n = 17,842 women) in 2011, 17,300 households (n = 17,863 women) in 2014, and 19,457 households (n = 20,127 women) in 2017–18.

### Study sample

A total of 17,736 women were encompassed in this study, comprising 5,479 women from 2011, 5,505 from 2014, and 6,752 women from the 2017–18 BDHS, as per the predetermined inclusion criteria. These inclusion criteria included being married or in a union, aged between 35 and 49 years, not primarily infertile, currently not being pregnant or within the postpartum amenorrhea period. We excluded women who want a baby within two years of the survey date.

### Outcome variable

The primary outcome for this study was the use of modern contraceptive methods. The relevant data was derived by asking two subsequent questions. Initially, eligible women were asked, *"Are you or your husband currently using any method to delay or avoid getting pregnant*?*"* The responses were reported dichotomously as either yes or no. If women provided an affirmative response, they were subsequently asked, *"Which method are you using*?*"* To answer this question, women were presented with a list of contraceptive methods’ names: pills, injections, implants, intrauterine devices (IUDs), condoms, female sterilization, male sterilization, periodic abstinence, and withdrawal. Additionally, an open option was provided if the contraception used was not listed. We reclassified these responses into two categories: "modern contraceptive methods users" and "others," in accordance with the World Health Organization’s classification [32]. Modern contraceptive methods include pills, injections, implants, IUDs, condoms, and female and male sterilization. Individuals who did not use any contraception or used traditional methods were collectively classified as "others."

### Explanatory variables

The year of the survey constituted a primary explanatory variable, aligned with the study’s objective. Other explanatory variables were selected based on a comprehensive literature search, as well as their availability in the analyzed surveys, and their statistical significance in relation to modern contraceptive methods uptake [4, 33–37]. The selected variables were women’s age (35–39, 40–44, 45–49 years), women’s education levels (no education, primary, secondary, higher), women’s employment status (unpaid work, paid work), and number of ever-born children (≤2 children, >2 children). Partner’s education attainment (no education, primary, secondary, higher), partner’s occupation categories (agriculture, physical worker, services, business, others), household type (nuclear (household member 4 or less), joint (household member 5 or more)), and wealth index were also included. The survey created wealth index variable through Principles Component Analysis (PCA) of the variables covering several households’ assets, including ownership radio, television and household’s roof type and reported it in the survey. The relevant procedure can be found in the respective survey reports [29–31]. Other variables included were place of residence (urban, rural), and region (Barisal, Chattogram, Dhaka, Khulna, Rajshahi, Rangpur, Sylhet).

### Statistical analysis

Descriptive analysis was used to explore the characteristics of study participants and the distribution of modern contraceptive method uptake across the explanatory variables considered. The association of each explanatory variable with the outcome variable was determined using the chi-square test. Multilevel logistic regression model was used to explore the associations of outcome variable with explanatory variables. The utilization of multilevel modeling stemmed from the hierarchical structure of the BDHS data, where individuals are nested within households and households are nested within communities. In this study design, an additional layer is introduced, representing the year of the survey. Previous studies have shown that for such data structures, multilevel modeling yields superior outcomes compared to conventional logistic regression models [38]. Multicollinearity was assessed before each analysis, and if evidence of multicollinearity was detected, relevant variables were removed. The outcomes are presented as adjusted Odds ratios (aOR) accompanied by their corresponding 95% confidence intervals (95% CI). All statistical analyses were conducted using Stata software version 14 (Stata Corp, College Station, Texas, USA).

### Ethics approval

The data analyzed in this study were obtained from the Demographic and Health Survey Program of the USA. Prior to conducting the survey in Bangladesh, approval was obtained from the institutional review board of ICF, USA, and subsequently from the National Research Ethics Committee of the Bangladesh Medical Research Council. To ensure the participants’ consent, informed written consent was acquired from all individuals involved, utilizing an appropriate institutional form. These consent forms were securely archived by the survey authority. In our research, we sought permission to access the data for analytical purposes, and the survey authority provided us with de-identified data. As the study involved secondary data analysis and adhered to the relevant guidelines and regulations, no additional ethical approval was required.

## Result

### Background characteristics of the respondents

Table 1 presents background characteristics of the respondents, while year-wise distribution is presented in S1 Table. The percentage of women within the later reproductive-aged cohort was found to be 37.4% during the 2011 BDHS, a figure that slightly increased to 38.6% and 39.6% during the BDHS conducted in 2014 and 2017–18, respectively. The cumulative proportion of these later reproductive-aged women was 38.7%. In 2011, 54.1% of later reproductive-aged women possessed an education attainment at or above the primary level. This percentage exhibited an incremental progression, reaching 57.6% and 68.8% in the 2014 and 2017–18 BDHS. Approximately three-quarters of the total women analyzed reported rural areas as their place of residence. Another noteworthy trend was the ascent in the percentage of women who reported engaging in paid employment, indicating a rapid increase across the triad of surveys–from 11.0% in 2011 to 38% in 2014, culminating at 56% in the 2017–18 iteration.

**Table 1:** Background characteristics of the later reproductive-aged women in Bangladesh, N = 17,736.

Characteristics	Overall	
	Participants Number (n)	Percentage (%)
**Women’s age (in years)**		
35–39	6,851	38.7
40–44	5,895	33.2
45–49	4,990	28.1
**Women’s education**		
No education	6,989	39.4
Primary	5,908	33.3
Secondary	3,694	20.8
Higher	1,145	6.5
**Women’s working status**		
Unpaid work	11,260	63.5
Paid work	6,476	36.5
**Women’s partner education**		
No education	6,429	36.3
Primary	4,810	27.2
Secondary	4,137	23.2
Higher	2,360	13.3
**Women’s partner occupation**		
Agriculture	6,203	35.0
Physical worker	5,400	30.4
Services	1,305	7.4
Business	3,839	21.6
Others	989	5.6
**Type of household**		
Nuclear (≤4)	7,491	42.3
Joint (>4)	10,245	57.7
**Number of children ever born**		
≤2 children	4,512	25.4
>2 children	13,224	74.6
**Wealth Index**		
Poorest	2,985	16.8
Poorer	3,609	20.3
Middle	3,662	20.7
Richer	3,572	20.1
Richest	3,908	22.1
**Media exposure**		
Unexposed	7,329	41.3
Exposed	10,407	58.7
**Place of residence**		
Urban	4,841	27.3
Rural	12,895	72.7
**Region**		
Barishal	1,120	6.3
Chattogram	2,997	16.9
Dhaka	5,563	31.4
Khulna	2,250	12.7
Rajshahi	2,051	11.6
Rangpur	2,300	12.9
Sylhet	1,455	8.2

**Note**: Established in 2015, the Mymensingh division was recognized as an independent region in the 2017–18 BDHS, following its previous inclusion in the Dhaka division during the 2011 and 2014 BDHS. To ensure uniform classification, we consequently reclassified the Mymensingh division under the Dhaka division for the 2017–18 BDHS.

### Distribution of modern contraceptive methods uptake

A noteworthy 61.83% (10,966) of women were identified as active users of various contraceptive methods, while the remaining 38.17% (6,770) chose not to partake in contraceptive practices as evidenced across the three surveys (Table 2). A breakdown reveals that 46.52% embraced modern contraceptive methods, whereas 15.31% favored traditional alternatives. Taking into account the comprehensive data, the overarching prevalence of modern contraceptive methods uptake stood at 46.52%. This figure demonstrated a consistent pattern with values of 46.09% in 2011, 45.97% in 2014, and 47.31% in the 2017–18 survey. Pills accounted for 19.13% of usage, followed by sterilization at 11.04%, and injections at 9.1%.

**Table 2:** Distribution of contraception use among later reproductive-aged women included in the Bangladesh Demographic and Health Survey, 2011 to 2017–28.

Contraceptive Use	2011 (N = 5,479) %	2014 (N = 5,505) %	2017–18 (N = 6,752) %	Total (N = 17,736) %
**Modern contraceptive method**	**46.09**	**45.97**	**47.33**	**46.52**
Pill	19.43	18.97	19.02	19.13
Injections	8.43	9.58	9.29	9.11
Condoms	4.61	4.99	5.60	5.11
Female sterilization*	10.03	8.32	9.05	9.13
Male sterilization*	1.80	2.06	1.89	1.91
Intra-uterine device (IUD)*	0.89	0.64	0.75	0.76
Implant*	0.90	1.41	1.70	1.36
**Traditional contraceptive method**	**15.36**	**13.73**	**16.55**	**15.31**
Periodic abstinence	12.36	11.05	13.26	12.29
Withdrawal	2.24	2.10	2.87	2.44
Others traditional methods	0.75	0.59	0.44	0.59
**Do not use contraception**	**38.55**	**40.30**	**36.12**	**38.17**
**Total**	**100.00**	**100.00**	**100.00**	**100.00**

^♀^ High effective contraceptive method

### Distribution of modern contraceptive methods use across respondents’ socio-demographic characteristics

**Fig 1:**
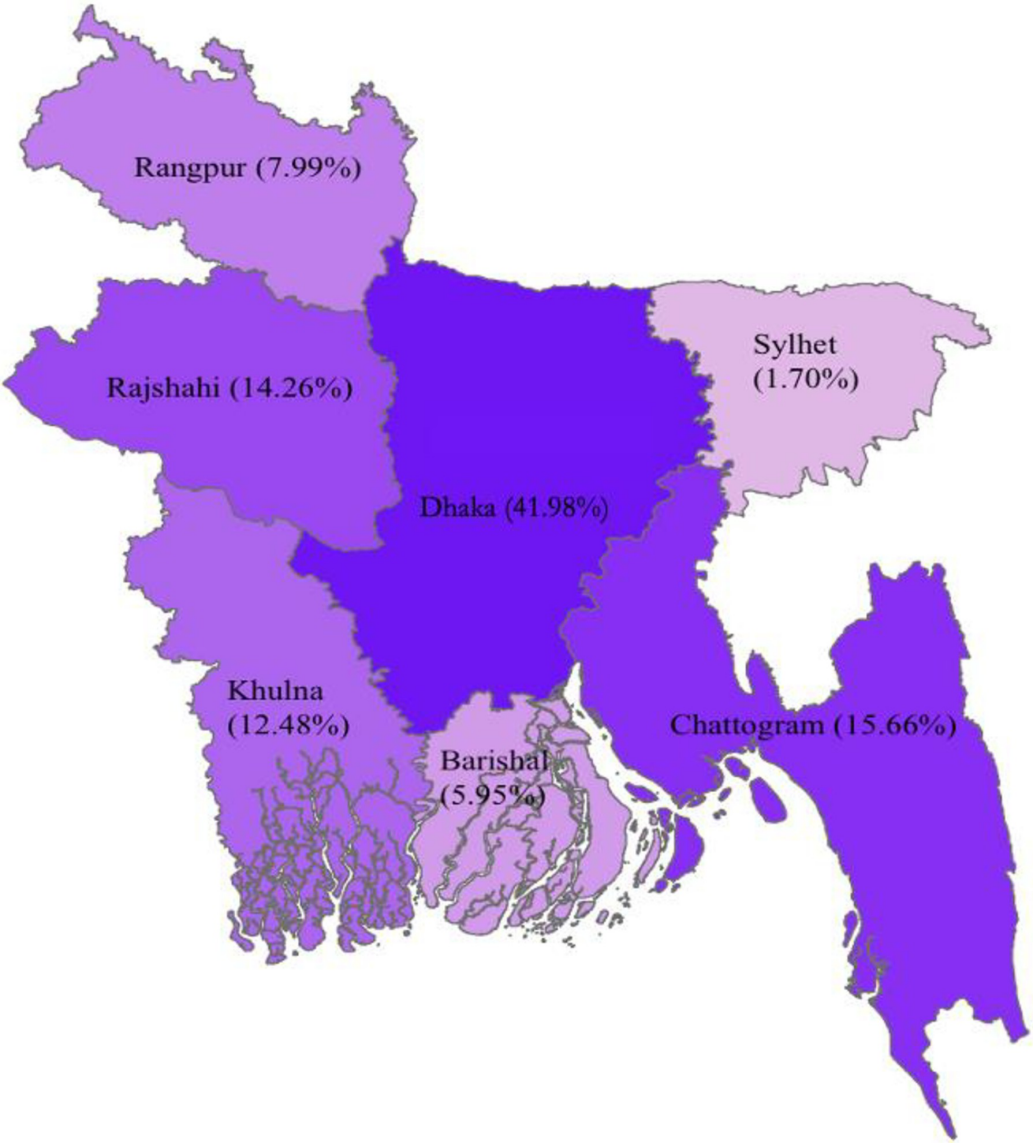
Regional distribution of modern contraceptive uptake among later reproductive-aged women in Bangladesh.

The overall distribution of the utilization of modern contraceptive methods across respondents’ socio-demographic characteristics is presented in Table 3, while year-wise distribution of modern contraceptive methods use is presented S2 Table. Women’s age, education background, occupational status, husband’s education and occupation, household type, and wealth index were identified as pertinent variables that intricately influenced the uptake of modern contraceptive methods. We also found significant regional level variations of modern contraceptive methods uptake among later reproductive-aged women in Bangladesh, ranging from 1.70% in Sylhet to 41.96% in Dhaka (Fig 1).

**Table 3:** Distribution of modern contraceptive method use among later reproductive-aged women included in the Bangladesh Demographic and Health Survey across their socio-demographic characteristics.

Characteristics	Modern contraceptive methods use status (n = 17,736)		p-value
	Use	Non-use	
**Women’s age (in years)**			p<0.001
35–39	50.22	28.55	
40–44	32.79	33.63	
45–49	16.99	37.82	
**Women’s education**			p<0.001
No education	37.84	40.77	
Primary	33.70	32.97	
Secondary	21.37	20.36	
Higher	7.09	5.90	
**Women’s working status**			p<0.001
Unpaid work	61.09	65.57	
Paid work	38.91	34.43	
**Women’s partner education**			p<0.001
No education	38.13	34.61	
Primary	26.86	27.35	
Secondary	21.82	24.63	
Higher	13.19	13.41	
**Women’s partner occupation**			p<0.001
Agriculture	35.93	34.14	
Physical worker	29.72	31.08	
Services	7.37	7.35	
Business	23.61	19.94	
Others	3.37	7.49	
**Type of Household**			p<0.001
Nuclear (≤4)	39.92	44.25	
Joint (>4)	60.08	55.75	
**No. of ever-born children**			p<0.01
≤2 children	24.13	26.58	
>2 children	75.87	73.42	
**Wealth Index**			p<0.001
Poorest	17.57	16.18	
Poorer	21.04	19.73	
Middle	21.27	20.10	
Richer	19.49	20.71	
Richest	20.61	23.27	
**Mass media exposure**			p<0.05
Unexposed	40.29	42.23	
Exposed	59.71	57.77	
**Place of residence**			p = 0.421
Urban	26.96	27.59	
Rural	73.04	72.41	
**Region of residence**			p<0.001
Barishal	5.95	6.63	
Chattogram	15.66	17.97	
Dhaka	41.96	39.93	
Khulna	12.48	12.87	
Rajshahi	14.26	11.85	
Rangpur	7.99	8.38	
Sylhet	1.70	2.37	

### Factors associated with modern contraceptive methods uptake among later reproductive-aged women in Bangladesh

The factors associated with the uptake of modern contraceptive methods were determined using the multilevel logistic regression model and the results are presented in Table 4. The results of each survey are presented in the S3 Table. We did not report a significant change in the likelihood of modern contraceptive methods uptake across the survey years. We found declined likelihoods of modern contraceptive method uptake among women aged 40–45 (aOR = 0.53, 95% CI: 0.49, 0.57) and 45–49 (aOR = 0.24, 95% CI: 0.22, 0.26) years old as compared to women aged 35–39 years old. In comparison to women with no education attainment, higher likelihoods (ranging from 112% to 142%) of modern contraceptive methods use were found among women with primary to higher education. An inverse association was observed for partner’s education, where an increasing level of partner’s education was negatively associated with the utilization of modern contraceptive methods. The likelihoods of modern contraceptive methods use were found 17% (0.83, 95% CI, 0.74–0.94) and 24% (aOR = 0.76, 95% CI: 0.66, 0.88) lower among women with richer and richest wealth quintile as compared to the women with poorest household wealth quintile. Higher likelihoods of modern contraceptive method use were found among women who residing in the Dhaka (aOR = 1.22, 95% CI: 1.07, 1.38) and Rajshahi (aOR = 1.37, 95% CI: 1.19, 1.59) regions compared to the women in the Barishal region. The likelihood of using modern contraceptive methods was found to be increased by 20% (aOR = 1.22; 95%CI: 1.13, 1.32) among those exposed to mass media, compared to those who were not exposed to mass media. Women who reported having paid work were 1.19 times more likely (aOR = 1.19; 95%CI: 1.10, 1.28) to uptake modern contraceptive methods as compared to the women who were not engaged in any paid work. The likelihood of modern contraceptive method uptake was found 43% higher (aOR, 1.43, 95% CI, 1.32–1.55) among women who had more than 2 children as compared to the women who had ≤2 children.

**Table 4:** Multilevel logistic regression model to explore likelihoods of modern contraceptive methods uptake across survey years adjusted for possible covariates, Bangladesh.

Characteristics	Modern contraceptive methods use		p-value
	aOR	95% CI	
**Year**			
2011	1.00		
2014	0.98	0.90–1.06	p = 0.641
2017	1.04	0.95–1.13	p = 0.405
**Women’s age (in years)**			
35–39	1.00		
40–44	0.53	0.49–0.57	p<0.001
45–49	0.24	0.22–0.26	p<0.001
**Women’s education**			
No education	1.00		
Primary	1.12	1.04–1.22	p<0.01
Secondary	1.17	1.05–1.30	p<0.01
Higher	1.42	1.18–1.70	p<0.001
**Women’s working status**			
Unpaid work	1.00		
Paid work	1.19	1.10–1.28	p<0.001
**Partner’s education**			
No education	1.00		
Primary	0.84	0.78–0.92	p<0.001
Secondary	0.80	0.72–0.88	p<0.001
Higher	0.80	0.69–0.93	p<0.01
**Partner’s occupation**			
Agriculture	1.00		
Physical worker	0.81	0.74–0.88	p<0.001
Services	0.99	0.85–1.15	p = 0.906
Business	1.11	1.01–1.22	p<0.05
Others	0.54	0.46–0.64	p<0.001
**Household types**			
Nuclear (≤4)	1.00		
Joint (>4)	1.13	1.06–1.22	p<0.001
**Number of ever-born children**			
≤2 children	1.00		
>2 children	1.43	1.32–1.55	p<0.001
**Wealth index**			
Poorest	1.00		
Poorer	0.98	0.88–1.08	p = 0.653
Middle	0.91	0.81–1.01	p = 0.089
Richer	0.83	0.74–0.94	p<0.01
Richest	0.76	0.66–0.88	p<0.001
**Mass media exposure**			
Unexposed	1.00		
Exposed	1.22	1.13–1.32	p<0.001
**Place of residence**			
Urban	1.00		
Rural	0.91	0.84–0.99	p<0.05
**Region**			
Barishal	1.00		
Chittagong	0.98	0.85–1.14	p = 0.821
Dhaka	1.22	1.07–1.38	p<0.01
Khulna	1.11	1.07–1.38	p = 0.171
Rajshahi	1.37	1.19–1.59	p<0.001
Rangpur	0.92	0.79–1.07	p = 0.268
Sylhet	0.77	0.63–0.95	p<0.05

## Discussion

The aim of this study was to determined the patterns of modern contraceptive methods uptake among later reproductive-aged women in Bangladesh. We also assessed how these trends changed over different survey periods and identified the factors that influence the uptake of modern contraceptive methods. No notable shifts in the uptake of modern contraceptive methods were observed over the surveyed years. The likelihood of adopting modern contraceptive methods exhibited a decline in correlation with the advancing age of women, as well as with the education level of women’s partners, and their inclusion within the richer or richest wealth quintile. Conversely, likelihoods of modern contraception uptake was found to be increased with higher levels of women’s education, increased exposure to mass media, and residence in either the Dhaka or Rajshahi division. These findings emphasize that the uptake of modern contraceptive methods remained relatively stable among later reproductive-aged women in Bangladesh, despite their increasing numbers over the years. In conjunction with the ongoing rise in the prevalence of pregnancies among later reproductive-aged women, this situation suggests that these women are at risk of experiencing unintended pregnancies and shorter birth intervals, both of which could lead to significant adverse consequences. It is crucial to link these observations with the current stagnation in the uptake of modern contraceptive methods in Bangladesh.

The findings of this study revealing a nearly 46% uptake of modern contraceptive methods among later reproductive-aged women, reflect concern for several reasons, despite the apparent improvement compared to other LMICs [5, 7, 25, 39–42]. The primary apprehension stems from the fact that this particular demographic currently constitutes more than 25% of the total reproductive-aged women in Bangladesh. Furthermore, this percentage is expected to increase in the forthcoming years due to the country’s population structure, where a significant number of women currently fall within the 15 to 30-year age range (38% of the total female population), and they will eventually transition into the 35+ cohort as time progresses [19, 26, 43]. Importantly, the inadequate increase in modern contraceptive methods use within this growing demographic should be considered one of the major factors contributing to the stagnation of contraception use rates in Bangladesh over the years [44].

Given the current pregnancy dynamics, it is notable that approximately one third of all pregnancies in Bangladesh are either unintended or occur within a short interval- a similar pattern was reported in other LMICs [1, 2, 7, 45]. Additionally, over half of these pregnancies involve women aged 35 or more [7, 46]. As the population of women aged 35 and above continues to grow, these numbers are poised to increase further in the coming years. Moreover, this challenge arises at a time when Bangladesh is experiencing an escalated prevalence of overweight (25.4%)/obesity (6.7%) alongside the existing burden of underweight among women aged 35 and more [47]. Chronic conditions, such as diabetes and hypertension, are also highly prevalent (40%) with later reproductive-aged women [22, 48]. A significant proportion of these cardiometabolic diseases remains undiagnosed, untreated, or uncontrolled in Bangladesh, with a notable increase current years [49, 50]. The convergence of unintended and short interval pregnancies with these chronic health conditions is anticipated to result in severe adverse consequences [6]. These consequences compound the existing burden of unintended and short interval pregnancies, leading to a reduced utilization of maternal healthcare services. This is frequently attributed to late pregnancy detection, dissatisfaction with the pregnancy, and reliance on prior pregnancy experiences. Collectively, these factors contribute to the heightened prevalence of adverse maternal and child health outcomes, including maternal and child mortality [3, 7]. Moreover, Bangladesh is currently experiencing a rapid shift in fertility age, with witnessing an increasing prevalence of pregnancies occurring after the age of 30 [46]. This trend is particularly conspicuous among higher-educated and urban women, within whom the prevalence of overweight/obesity status and chronic conditions is also notably high. Collectively, these findings underscore a serious challenge that Bangladesh will face in the coming years if proactive measures to ensure contraception among this growing demographic are not implemented [51].

The consistent rate of modern contraceptive method use among later reproductive-aged women is likely due to a prevailing focus solely on earlier reproductive-aged women [20]. This tendency primarily arises from the misconception within communities that contraception is primarily necessary during the initial phases of reproductive life [51]. This oversight at the service providers’ level is often accompanied by challenges faced by comparatively later reproductive-aged women, including a sense of discomfort when seeking contraception [52]. Furthermore, women in this age group often lead busy lives, juggling family responsibilities and occupations, which can lead to contraception being perceived as a lower-priority issue. Adding to this, a notable proportion of these women also manage chronic conditions, potentially leading to the misconception that such health issues influence their fertility to the extent that contraception becomes unnecessary [4]. This specific misconception is widespread in Bangladesh, particularly among those who are illiterate and homemakers, and it contributes to the prevailing dynamics within this demographic [22, 53]. Supporting this notion, our study reported higher likelihoods of modern contraceptive method usage among women with comparatively higher education, formal occupations and higher exposure to mass media that increase knowledge about importance of using contraception, similar to other available studies [4, 41].

This study has also reported lower likelihoods of modern contraception uptake as the level of women’s partner education rises which contradicts with a previous observation for earlier reproductive-aged women [41]. This dynamic is reported alongside the traditional existence in Bangladesh, where educated partners have typically educated wives, and wives are the primary users of contraception, with husbands exerting a major influence on the decision-making process [15, 54]. Together, this suggests a decline in the importance of contraception among husbands as women’s age increases, which subsequently affects the overall uptake of contraception in later reproductive-aged women.

The findings regarding regional variations in contraception uptake align with the overall pattern in Bangladesh [29–31]. These differences are likely associated with factors such as healthcare facility density, the quality of family planning services, prevailing social norms, and variations in education at the regional level [55]. Another significant factor may be the differences in the coverage of rurality across divisions. For example, Sylhet division, with a higher proportion of rural areas, showed lower likelihoods of modern contraception uptake in this study. In contrast, Dhaka, Khulna, and Rajshahi divisions, with higher coverage of urban areas, reported higher uptake of modern contraception methods.

This study has several strengths and a few limitations. Analysis of this study covering multiple survey rounds provides a comprehensive view of how modern contraceptive utilization has evolved over 2011–2018 among later reproductive-aged women in Bangladesh. The use of nationally representative data enhances the generalizability of the findings to the broader population of later reproductive-aged women in the country, while also ensuring a diverse geographic representation that strengthens the robustness of the results. The study employs a multilevel logistic regression model to explore the associations between various socio-demographic factors and modern contraceptive use among later reproductive-aged women. This approach helps to account for hierarchical data structures within the BDHS datasets, thereby improving the accuracy of the estimates regarding the factors that influence modern contraceptive uptake. By including a range of socio-demographic characteristics, the study offers a comprehensive understanding of the multifaceted determinants that shape contraceptive choices among this demographic [26]. However, certain limitations should be acknowledged when interpreting the study findings [56]. BDHS data are self-reported- and as such, they may introduce recall bias. Additionally, the cross-sectional nature of the BDHS data restricts the study’s ability to establish causal relationships; while associations can be identified, causation cannot be inferred. The analysis is constrained by the available variables in the BDHS data sets, potentially omitting relevant contextual factors that influence contraceptive decision-making [15]. Furthermore, the study’s findings could not capture the evolving landscape of healthcare access, family planning programs, and societal attitudes towards contraception because of the lack of relevant variables in the data sets [51]. Despite these limitations, the study provides valuable insights into the trends and determinants of modern contraceptive uptake among later reproductive-aged women in Bangladesh, offering a foundation for future research and policy considerations.

## Conclusion

Nearly 54% of women in Bangladesh aged 35 and more do not use modern contraceptive methods, with no significant shifts observed over the surveyed years. The likelihood of using modern contraceptive methods declines notably with increasing age, partner’s education level, and wealth quintile. Conversely, an increased likelihood of embracing modern contraceptive methods was observed among women with higher education, increased exposure to mass media, and residence in Dhaka or Rajshahi division. These findings highlight the stable uptake of modern contraceptive methods among women aged 35 or more, despite their growing representation in the population. The persistent trend of stagnation calls for proactive measures to address the specific needs of later reproductive-aged women in family planning programs. Strengthening awareness campaigns, improving healthcare access, and tailoring interventions could lead to a more effective and responsive approach to contraception among later reproductive-aged women.

## Supporting information

S1 Table Background characteristics of the late reproductive-aged women, Bangladesh. (DOCX)

S2 Table Distribution of later reproductive-aged women reported their current modern contraceptive method use patterns in different BDHS. (DOCX)

S3 Table Multilevel logistic regression model to explore likelihoods of modern contraceptive methods use across survey years adjusted for possible covariates, Bangladesh. (DOCX)

S1 Checklist STROBE statement—Checklist of items that should be included in reports of observational studies. (DOCX)

## Abbreviations

aOR: Adjusted Odds Ratio
BDHS: Bangladesh Demographic and Health Survey
CI: Confidence Interval
IUD: Intrauterine Device
LMIC: Low- and Middle-Income Country
SDG: Sustainable Development Goals
