# Correction: Exploration of modern contraceptive methods using patterns among later reproductive-aged women in Bangladesh

**DOI:** 10.1371/journal.pone.0305488

**Published:** 2024-06-11

**Authors:** Md. Shohel Rana, Shimlin Jahan Khanam, Md. Badsha Alam, Tahir Ahmed Hassen, Md. Iqbal Kabir, Md. Nuruzzaman Khan

The fourth author’s name is spelled incorrectly. The correct name is: Tahir Ahmed Hassen. The correct citation is: Rana MS, Khanam SJ, Alam MB, Hassen TA, Kabir MI, Khan MN (2024) Exploration of modern contraceptive methods using patterns among later reproductive-aged women in Bangladesh. PLoS ONE 19(4): e0291100. https://doi.org/10.1371/journal.pone.0291100
