# Supplementary material for: Exploration of modern contraceptive methods using patterns among later reproductive-aged women in Bangladesh
Source: PLoS One. 2024 Apr 1;19(4):e0291100. doi: 10.1371/journal.pone.0291100 (PMC10984413; doi:10.1371/journal.pone.0291100)
Supplement: S1 Table — (DOCX) [file pone.0291100.s001.docx]

**Supplementary Table 1. Background characteristics of the late reproductive-aged women, Bangladesh.**

| **Characteristics** | **2011**  **(n=5,479)** | | **2014**  **(n=5,505)** | | **2017-18 (n=6,752)** | | **Overall**  **(n=17,736)** | |
| --- | --- | --- | --- | --- | --- | --- | --- | --- |
| **Women’s age** | N | % | N | % | N | % | N | % |
| Age 35-39 | 2,052 | 37.4 | 2,125 | 38.6 | 2,675 | 39.6 | 6,851 | 38.7 |
| Age 40-44 | 1,931 | 35.2 | 1,868 | 33.9 | 2,096 | 31.0 | 5,895 | 33.2 |
| Age 45-49 | 1,496 | 27.4 | 1,512 | 27.5 | 1,981 | 29.4 | 4,990 | 28.1 |
| **Women’s education** |  |  |  |  |  |  |  |  |
| No education | 2,512 | 45.9 | 2,333 | 42.4 | 2,143 | 31.7 | 6,989 | 39.4 |
| Primary | 1,635 | 29.9 | 1,722 | 31.3 | 2,552 | 37.8 | 5,908 | 33.3 |
| Secondary | 1,002 | 18.2 | 1,135 | 20.6 | 1,558 | 23.1 | 3,694 | 20.8 |
| Higher | 330 | 6.0 | 315 | 5.7 | 499 | 7.4 | 1,145 | 6.5 |
| **Women’s working status** |  |  |  |  |  |  |  |  |
| Unpaid work | 4,855 | 88.6 | 3,431 | 62.3 | 2,975 | 44.1 | 11,260 | 63.5 |
| Paid work | 624 | 11.4 | 2,074 | 37.7 | 3,777 | 55.9 | 6,476 | 36.5 |
| **Women’s partner education** |  |  |  |  |  |  |  |  |
| No education | 2,082 | 38.0 | 2,086 | 37.9 | 2,260 | 33.5 | 6,429 | 36.3 |
| Primary | 1,384 | 25.3 | 1,364 | 24.8 | 2,061 | 30.5 | 4,810 | 27.2 |
| Secondary | 1,257 | 22.9 | 1,317 | 23.9 | 1,563 | 23.1 | 4,137 | 23.2 |
| Higher | 756 | 13.8 | 738 | 13.4 | 868 | 12.9 | 2,360 | 13.3 |
| **Women’s partner occupation** |  |  |  |  |  |  |  |  |
| Agriculture | 1,934 | 35.2 | 1,862 | 33.8 | 2,407 | 35.7 | 6,203 | 35.0 |
| Physical worker | 1,477 | 26.9 | 1,673 | 30.4 | 2,250 | 33.4 | 5,400 | 30.4 |
| Services | 505 | 9.2 | 439 | 8.0 | 362 | 5.4 | 1,305 | 7.4 |
| Business | 1,205 | 22.0 | 1,237 | 22.5 | 1,396 | 20.7 | 3,839 | 21.6 |
| Others | 358 | 6.7 | 294 | 5.3 | 337 | 4.8 | 989 | 5.6 |
| **Type of household** |  |  |  |  |  |  |  |  |
| Nuclear (≤4) | 2,032 | 37.1 | 2,328 | 42.3 | 3,132 | 46.4 | 7,491 | 42.3 |
| Joint (>4) | 3,447 | 62.9 | 3,177 | 57.7 | 3,620 | 53.6 | 10,245 | 57.7 |
| **Number of children ever born** |  |  |  |  |  |  |  |  |
| ≤2 children | 1,226 | 22.4 | 1,407 | 25.5 | 1,878 | 27.8 | 4,512 | 25.4 |
| >2 children | 4,253 | 77.6 | 4,098 | 74.5 | 4,874 | 72.2 | 13,224 | 74.6 |
| **Wealth Index** |  |  |  |  |  |  |  |  |
| Poorest | 880 | 16.1 | 927 | 17.1 | 1,177 | 17.5 | 2,985 | 16.8 |
| Poorer | 1,087 | 19.8 | 1,130 | 20.5 | 1,392 | 20.6 | 3,609 | 20.3 |
| Middle | 1,141 | 20.9 | 1,117 | 20.2 | 1,405 | 20.9 | 3,662 | 20.7 |
| Richer | 1,110 | 20.2 | 1,124 | 20.3 | 1,338 | 19.8 | 3,572 | 20.1 |
| Richest | 1,261 | 23.0 | 1,207 | 21.9 | 1,440 | 21.2 | 3,908 | 22.1 |
| **Media exposure** |  |  |  |  |  |  |  |  |
| Unexposed | 2,292 | 41.9 | 2,366 | 43.0 | 2.671 | 39.6 | 7,329 | 41.3 |
| Exposed | 3,187 | 58.1 | 3,139 | 57.0 | 4,081 | 60.4 | 10,407 | 58.7 |
| **Place of residence** |  |  |  |  |  |  |  |  |
| Urban | 1,456 | 26.5 | 1,550 | 28.2 | 1,835 | 27.2 | 4,841 | 27.3 |
| Rural | 4,023 | 73.5 | 3,955 | 71.8 | 4,917 | 72.8 | 12,895 | 72.7 |
| **Region of residence** |  |  |  |  |  |  |  |  |
| Barisal | 327 | 6.0 | 379 | 6.8 | 414 | 6.1 | 1,120 | 6.3 |
| Chittagong | 918 | 16.8 | 943 | 17.1 | 1,136 | 16.8 | 2,997 | 16.9 |
| Dhaka | 1,764 | 32.2 | 1,848 | 33.6 | 2,099 | 31.1 | 5,563 | 31.4 |
| Khulna | 718 | 13.1 | 644 | 11.7 | 887 | 13.1 | 2,250 | 12.7 |
| Rajshahi | 839 | 15.3 | 699 | 12.7 | 1,029 | 15.3 | 2,051 | 11.6 |
| Rangpur | 630 | 11.5 | 642 | 11.7 | 823 | 12.2 | 2,300 | 12.9 |
| Sylhet | 282 | 5.1 | 350 | 6.4 | 364 | 5.4 | 1,455 | 8.2 |
