# Supplementary material for: Exploration of modern contraceptive methods using patterns among later reproductive-aged women in Bangladesh
Source: PLoS One. 2024 Apr 1;19(4):e0291100. doi: 10.1371/journal.pone.0291100 (PMC10984413; doi:10.1371/journal.pone.0291100)
Supplement: S2 Table — (DOCX) [file pone.0291100.s002.docx]

**Supplementary Table 2: Distribution of later reproductive-aged women reported their current modern contraceptive method use patterns in different BDHS.**

| **Characteristics** | **Modern contraceptive methods use status** | | | | | | | | | | | |
| --- | --- | --- | --- | --- | --- | --- | --- | --- | --- | --- | --- | --- |
|  | **2011** | | | **2014** | | | **2017-18** | | | **Overall** | | |
|  | **Use** | **Non-use** | **P-value** | **Use** | **Non-use** | **P-value** | **Use** | **Non-use** | **p-value** | **Use** | **Non-use** | **p-value** |
| **Women’s age** |  |  |  |  |  |  |  |  |  |  |  |  |
| Age 35-39 | 46.80 | 29.45 | p<0.001 | 51.56 | 27.56 | p<0.001 | 51.86 | 28.63 | p<0.001 | 50.22 | 28.55 | p<0.001 |
| Age 40-44 | 35.21 | 35.28 |  | 33.49 | 34.31 |  | 30.32 | 31.68 |  | 32.79 | 33.63 |  |
| Age 45-49 | 17.99 | 35.27 |  | 14.95 | 38.13 |  | 17.82 | 39.69 |  | 16.99 | 37.82 |  |
| **Women’s education** |  |  |  |  |  |  |  |  |  |  |  |  |
| No education | 45.65 | 46.02 | p<0.05 | 40.45 | 44.03 | p=0.147 | 29.59 | 33.67 | p<0.01 | 37.84 | 40.77 | p<0.01 |
| Primary | 29.18 | 30.38 |  | 32.25 | 30.46 |  | 38.44 | 37.21 |  | 33.70 | 32.97 |  |
| Secondary | 18.04 | 18.50 |  | 21.03 | 20.24 |  | 24.26 | 22.02 |  | 21.37 | 20.36 |  |
| Higher | 7.13 | 5.10 |  | 6.27 | 5.27 |  | 7.71 | 7.10 |  | 7.09 | 5.90 |  |
| **Women’s working status** |  |  |  |  |  |  |  |  |  |  |  |  |
| Unpaid work | 88.12 | 89.02 | p=383 | 60.54 | 63.83 | p<0.05 | 40.15 | 47.56 | p<0.001 | 61.09 | 65.57 | p<0.001 |
| Paid work | 11.88 | 10.98 |  | 39.46 | 36.17 |  | 59.85 | 52.44 |  | 38.91 | 34.43 |  |
| **Women’s partner education** |  |  |  |  |  |  |  |  |  |  |  |  |
| No education | 40.45 | 35.9 | p<0.001 | 39.83 | 36.26 | p=247 | 34.94 | 32.15 | p<0.05 | 38.13 | 34.61 | p<0.01 |
| Primary | 23.83 | 26.49 |  | 24.62 | 24.91 |  | 31.02 | 30.09 |  | 26.86 | 27.35 |  |
| Secondary | 21.11 | 24.50 |  | 22.39 | 25.24 |  | 21.93 | 24.24 |  | 21.82 | 24.63 |  |
| Higher | 14.61 | 13.11 |  | 13.16 | 13.59 |  | 12.11 | 13.52 |  | 13.19 | 13.41 |  |
| **Women’s partner occupation** |  |  |  |  |  |  |  |  |  |  |  |  |
| Agriculture | 38.10 | 32.89 | p<0.001 | 31.19 | 36.07 | p<0.01 | 37.97 | 33.56 | p<0.001 | 35.93 | 34.14 | p<0.001 |
| Physical worker | 24.95 | 28.67 |  | 32.19 | 28.86 |  | 31.53 | 34.94 |  | 29.72 | 31.08 |  |
| Services | 9.22 | 9.22 |  | 8.04 | 7.92 |  | 5.38 | 5.34 |  | 7.37 | 7.35 |  |
| Business | 24.04 | 20.26 |  | 24.83 | 20.47 |  | 22.3 | 19.23 |  | 23.61 | 19.94 |  |
| Others | 3.69 | 8.96 |  | 3.75 | 6.68 |  | 2.80 | 6.93 |  | 3.37 | 7.49 |  |
| **Type of Household** |  |  |  |  |  |  |  |  |  |  |  |  |
| Nuclear (≤4) | 35.97 | 38.03 | p=191 | 39.64 | 44.52 | p<0.01 | 43.27 | 49.19 | p<0.001 | 39.92 | 44.25 | p<0.001 |
| Joint (>4) | 64.03 | 61.97 |  | 60.36 | 55.48 |  | 56.73 | 50.81 |  | 60.08 | 55.75 |  |
| **No. of ever-born children** |  |  |  |  |  |  |  |  |  |  |  |  |
| ≤2 children | 20.79 | 23.75 | p<0.01 | 25.05 | 26 | P=0.527 | 26.05 | 29.41 | p<0.01 | 24.13 | 26.58 | p<0.01 |
| >2 children | 79.21 | 76.25 |  | 74.95 | 74 |  | 73.95 | 70.59 |  | 75.87 | 73.42 |  |
| **Wealth Index** |  |  |  |  |  |  |  |  |  |  |  |  |
| Poorest | 16.48 | 15.69 | p=0.088 | 17.13 | 16.61 | p=0.656 | 18.77 | 16.24 | p<0.01 | 17.57 | 16.18 | p<0.01 |
| Poorer | 21.05 | 18.81 |  | 20.87 | 20.25 |  | 21.22 | 20.07 |  | 21.04 | 19.73 |  |
| Middle | 21.35 | 20.39 |  | 21.08 | 19.58 |  | 21.37 | 20.39 |  | 21.27 | 20.10 |  |
| Richer | 19.65 | 20.78 |  | 19.72 | 21.03 |  | 19.18 | 20.39 |  | 19.49 | 20.71 |  |
| Richest | 21.48 | 24.33 |  | 21.20 | 22.54 |  | 19.46 | 23.01 |  | 20.61 | 23.27 |  |
| **Mass media exposure** |  |  |  |  |  |  |  |  |  |  |  |  |
| Unexposed | 40.7 | 42.81 | P=0.162 | 42.37 | 43.5 | p=0.499 | 38.31 | 40.68 | p=080 | 40.29 | 42.23 | p<0.05 |
| Exposed | 59.3 | 57.19 |  | 57.63 | 56.5 |  | 61.69 | 59.32 |  | 59.71 | 57.77 |  |
| **Place of residence** |  |  |  |  |  |  |  |  |  |  |  |  |
| Urban | 25.77 | 27.27 | p=283 | 28.19 | 28.13 | p=0.970 | 26.91 | 27.41 | p=0.686 | 26.96 | 27.59 | p=421 |
| Rural | 74.23 | 72.73 |  | 71.81 | 71.87 |  | 73.09 | 72.59 |  | 73.04 | 72.41 |  |
| **Region of residence** |  |  |  |  |  |  |  |  |  |  |  |  |
| Barisal | 6.30 | 5.58 | p<0.001 | 6.72 | 7.01 | p<0.05 | 5.63 | 6.58 | p<0.001 | 5.95 | 6.63 | p<0.001 |
| Chattogram | 17.20 | 16.32 |  | 16.32 | 17.83 |  | 14.70 | 18.72 |  | 15.66 | 17.97 |  |
| Dhaka | 32.82 | 31.48 |  | 33.80 | 33.38 |  | 32.28 | 30.00 |  | 41.96 | 39.93 |  |
| Khulna | 12.94 | 13.33 |  | 11.33 | 12.03 |  | 12.72 | 13.52 |  | 12.48 | 12.87 |  |
| Rajshahi | 13.66 | 17.27 |  | 13.60 | 11.93 |  | 16.61 | 14.01 |  | 14.26 | 11.85 |  |
| Rangpur | 10.47 | 12.70 |  | 12.84 | 10.65 |  | 13.66 | 10.87 |  | 7.99 | 8.38 |  |
| Sylhet | 6.61 | 3.42 |  | 5.39 | 7.17 |  | 4.40 | 6.30 |  | 1.70 | 2.37 |  |
