# Supplementary material for: Exploration of modern contraceptive methods using patterns among later reproductive-aged women in Bangladesh
Source: PLoS One. 2024 Apr 1;19(4):e0291100. doi: 10.1371/journal.pone.0291100 (PMC10984413; doi:10.1371/journal.pone.0291100)
Supplement: S3 Table — (DOCX) [file pone.0291100.s003.docx]

**Supplementary Table 3: Multilevel logistic regression model to explore likelihoods of modern contraceptive methods use across survey years adjusted for possible covariates, Bangladesh.**

| Characteristics | **2011** | **2014** | **2017-18** | **Overall** |
| --- | --- | --- | --- | --- |
|  | aOR (95% CI) | aOR (95% CI) | aOR (95% CI) | aOR (95% CI) |
| **Year** |  |  |  |  |
| 2011 | N/A | N/A | N/A | 1.00 |
| 2014 | N/A | N/A | N/A | 0.98 (0.90-1.06) |
| 2017 | N/A | N/A | N/A | 1.04 (0.95-1.13) |
| **Women’s age** |  |  |  |  |
| Age 35-39 | 1.00 | 1.00 | 1.00 | 1.00 |
| Age 40-44 | 0.58 (0.51-0.67) ^***^ | 0.47 (0.42-0.54) ^***^ | 0.51 (0.46-0.58) ^***^ | 0.53 (0.49-0.57) ^***^ |
| Age 45-49 | 0.29 (0.25-0.34) ^***^ | 0.19 (0.16-0.22) ^***^ | 0.22 (0.19-0.26) ^***^ | 0.24 (0.22-0.26) ^***^ |
| **Women’s education** |  |  |  |  |
| No education | 1.00 | 1.00 | 1.00 | 1.00 |
| Primary | 1.04 (0.90-1.21) | 1.18 (1.01-1.37) ** | 1.19 (1.04-1.36) ** | 1.12 (1.04-1.22) ^***^ |
| Secondary | 1.13 (0.91-1.39) | 1.09 (0.89-1.33) | 1.34 (1.12-1.61) ^***^ | 1.17 (1.05-1.30) ^***^ |
| Higher | 1.68 (1.18-2.37) ^**^ | 1.40 (1.00-1.97) ** | 1.39 (1.05-1.86) ^**^ | 1.42 (1.18-1.70) ^***^ |
| **Women’s working status** |  |  |  |  |
| Unpaid work | 1.00 | 1.00 | 1.00 | 1.00 |
| Paid work | 1.06 (0.88-1.27) | 1.17 (1.03-1.32) ^**^ | 1.23 (1.10-1.38) ^***^ | 1.19 (1.10-1.28) ^***^ |
| **Partner’s education** |  |  |  |  |
| No education | 1.00 | 1.00 | 1.00 | 1.00 |
| Primary | 0.83 (0.71-0.98) ^**^ | 0.81 (0.69-0.96) ^**^ | 0.84 (0.69-0.91) ^**^ | 0.84 (0.78-0.92) ^***^ |
| Secondary | 0.77 (0.64-0.93) ^***^ | 0.80 (0.67-0.97) ^**^ | 0.78 (0.66-0.92) ^***^ | 0.80 (0.72-0.88) ^***^ |
| Higher | 0.80 (0.60-1.07) | 0.73 (0.54-0.97) ^**^ | 0.77 (0.60-0.99) ^**^ | 0.80 (0.69-0.93) ^***^ |
| **Partner’s occupation** |  |  |  |  |
| Agriculture | 1.00 | 1.00 | 1.00 | 1.00 |
| Physical worker | 0.71 (0.61-0.84) | 0.99 (0.85-1.16) | 0.79 (0.69-0.91) ^***^ | 0.81 (0.74-0.88) ^***^ |
| Services | 0.88 (0.68-1.14) ^**^ | 1.23 (0.92-1.64) | 0.95 (0.72-1.24) | 0.99 (0.85-1.15) |
| Business | 1.07 (0.91-1.28) ^***^ | 1.30 (1.10-1.54) ^***^ | 1.05 (0.90-1.23) | 1.11 (1.01-1.22) ^**^ |
| Others | 0.46 (0.35-0.60) | 0.73 (0.54-0.99) | 0.51 (0.39-0.68) ^***^ | 0.54 (0.46-0.64) ^***^ |
| **Household types** |  |  |  |  |
| Nuclear (≤4) | 1.00 | 1.00 | 1.00 | 1.00 |
| Joint (>4) | 1.09 (1.25-1.71) ^**^ | 1.23 (1.08-1.39) ^***^ | 1.20 (1.07-1.34) ^***^ | 1.13 (1.06-1.22) ^***^ |
| **Number of ever-born children** |  |  |  |  |
| ≤2 children | 1.00 | 1.00 | 1.00 | 1.00 |
| >2 children | 1.46 (1.25-1.71) ^**^ | 1.46 (1.26-1.70) ^***^ | 1.47 (1.29-1.67) ^***^ | 1.43 (1.32-1.55) ^***^ |
| **Wealth index** |  |  |  |  |
| Poorest | 1.00 | 1.00 | 1.00 | 1.00 |
| Poorer | 1.02 (0.89-1.23) | 1.00 (0.82-1.21) | 0.94 (0.79-1.12) | 0.98 (0.88-1.08) |
| Middle | 0.89 (0.73-1.10) | 0.84 (0.68-1.04) | 1.02 (0.84-1.23) | 0.91 (0.81-1.01) |
| Richer | 0.82 (0.66-1.03) | 0.84 (0.67-1.06) | 0.87 (0.71-1.07) | 0.83 (0.74-0.94) ^***^ |
| Richest | 0.77 (0.59-1.01) | 0.74 (0.57-0.98) ^**^ | 0.85 (0.67-1.07) | 0.76 (0.66-0.88) ^***^ |
| **Mass media exposure** |  |  |  |  |
| Unexposed | 1.00 | 1.00 | 1.00 | 1.00 |
| Exposed | 1.30 (1.12-1.50) ^***^ | 1.18 (1.01-1.37) ^**^ | 1.19 (1.04-1.35) ^***^ | 1.22 (1.13-1.32) ^***^ |
| **Place of residence** |  |  |  |  |
| Urban | 1.00 | 1.00 | 1.00 | 1.00 |
| Rural | 0.92 (0.78-1.10) | 0.89 (0.76-1.04) | 0.91 (0.79-1.05) | 0.91 (0.84-0.99) |
| **Region of residence** |  |  |  |  |
| Barisal | 1.00 | 1.00 | 1.00 | 1.00 |
| Chittagong | 1.06 (0.81-1.39) | 0.92 (0.71-1.18) | 0.91 (0.71-1.15) | 0.98 (0.85-1.14) |
| Dhaka | 1.04 (0.80-1.35) | 1.07 (0.83-1.37) | 1.31 (1.06-1.62) ^**^ | 1.22 (1.07-1.38) ^***^ |
| Khulna | 1.12 (0.86-1.45) | 1.09 (0.85-1.40) | 1.06 (0.84-1.35) | 1.11 (1.07-1.38) |
| Rajshahi | 1.37 (1.05-1.79) ^**^ | 1.27 (0.98-1.64) ^**^ | 1.49(1.18-1.90) ^***^ | 1.37 (1.19-1.59) ^***^ |
| Rangpur | 1.32 (1.00-1.73) ^**^ | 1.41 (1.09-1.82) ^**^ | 1.43 (1.12-1.82) ^***^ | 0.92 (0.79-1.07) |
| Sylhet | 0.59 (0.44-1.79) | 0.73 (0.56-0.97) | 0.81 (0.63-1.05) | 0.77 (0.63-0.95) ^**^ |
